# Supplementary material for: Spin-current probe for phase transition in an insulator
Source: Nat Commun. 2016 Aug 30;7:12670. doi: 10.1038/ncomms12670 (PMC5013713; doi:10.1038/ncomms12670)
Supplement: Supplementary Information — Supplementary Figures 1-4, Supplementary Notes 1-3 and Supplementary References [file ncomms12670-s1.pdf]

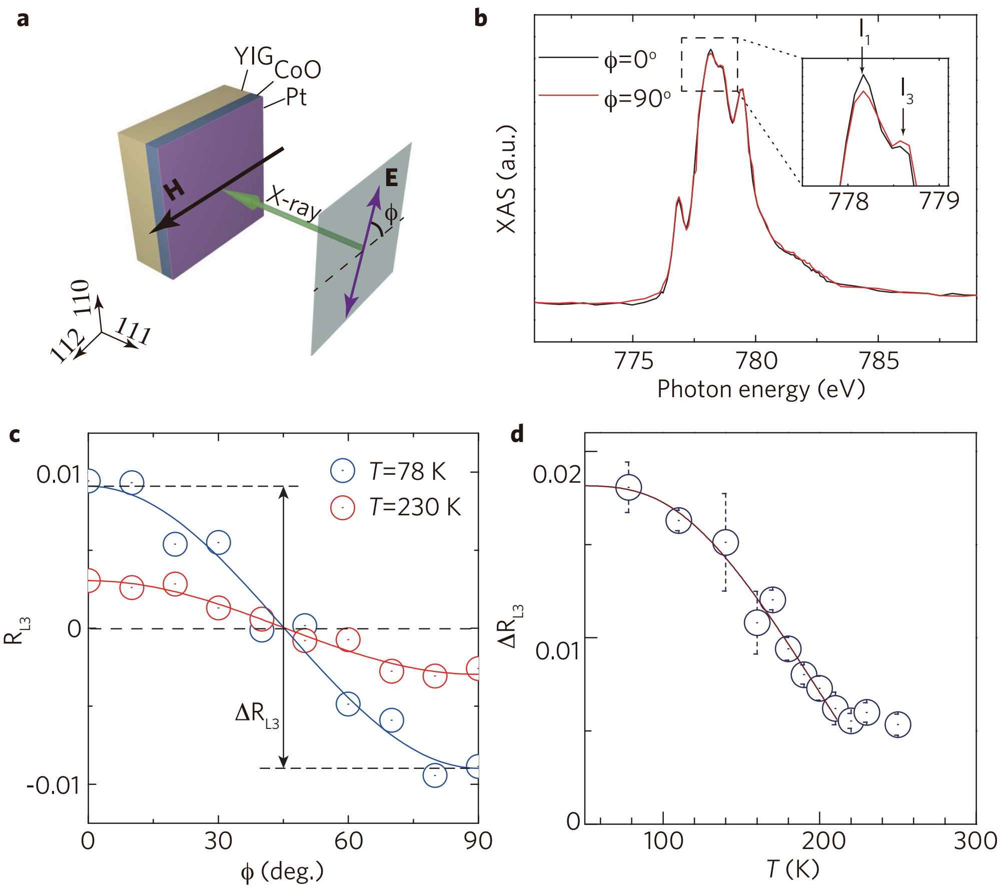

Supplementary Figure 1: **a**. Illustration of the geometry for the XMLD measurement. **b**. XAS of CoO is recorded at 78 K with two different x-ray polarizations (along [112] axis and [110] axis). **c**. R_{L3} is recorded at 78 K and 230 K with x-ray linear polarization turning from $\phi = 0°$ to $90°$. The data is well fitted by $R_{L3} = A\cos^2\phi + B$ (solid curves). **d**. ΔR_{L3} as a function of temperature. The error bars represent the s.d. of multiple measurements at the same condition.

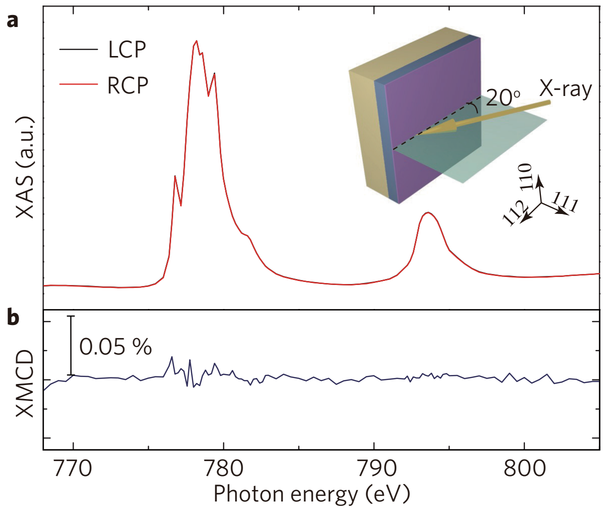

Supplementary Figure 2: **a**. x-ray absorption spectroscopy (XAS) and x-Ray magnetic circular dichroism (XMCD) signals for CoO in the $Y_3Fe_5O_{12}$/CoO (6 nm)/Pt (1 nm) film. LCP and RCP refer to left circular polarization and right circular polarization, respectively.

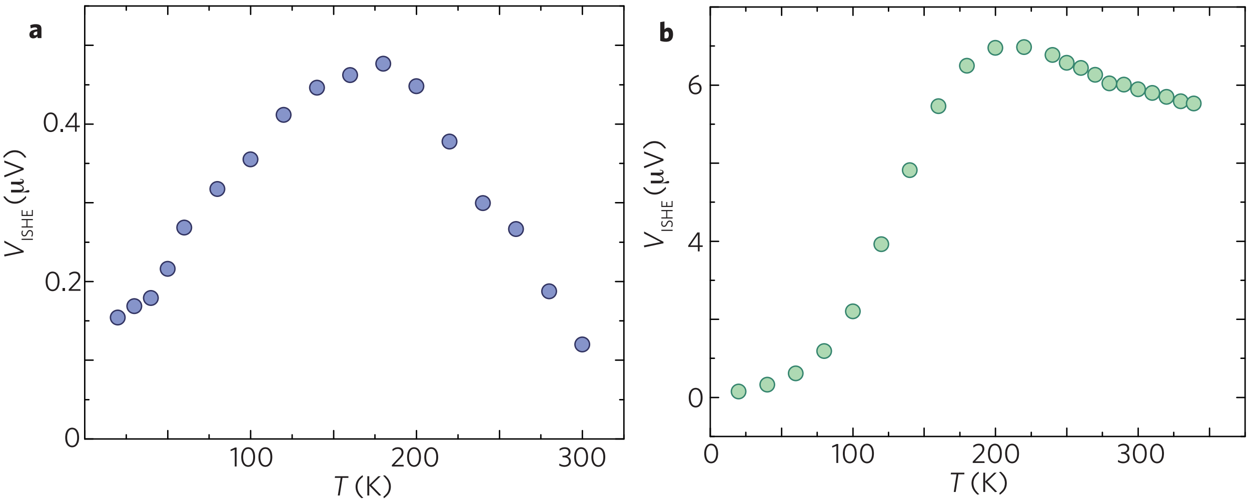

Supplementary Figure 3: Temperature dependence of V_{ISHE} for the $Y_3Fe_5O_{12}$ (3 μm)/Cu (5 nm)/CoO (6 nm)/Pt (10 nm) film (**a**) and the $Y_3Fe_5O_{12}$ (3 μm)/Cu (5 nm)/NiO (1.5 nm)/Pt (10 nm) film (**b**).

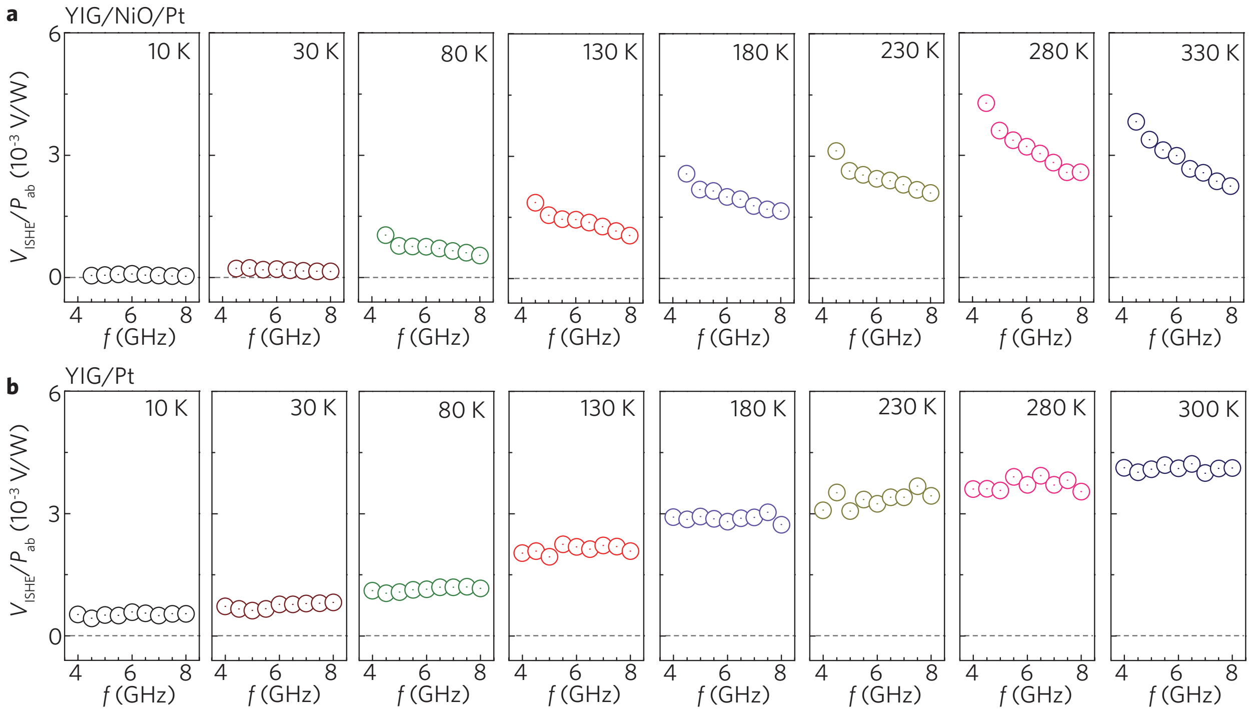

Supplementary Figure 4: Frequency dependence of $V_{\mathrm{ISHE}}/P_{\mathrm{ab}}$ at various temperatures for the $Y_3Fe_5O_{12}$ (3 μm)/NiO (1.5 nm)/Pt (10 nm) trilayer film (**a**) and the $Y_3Fe_5O_{12}$ (3 μm)/Pt (10 nm) bilayer film (**b**).

Supplementary Note 1: XMLD and XMCD measurements

$Y_3Fe_5O_{12}$/CoO (6 nm)/Pt (1 nm) was studied by x-ray Magnetic Linear Dichroism (XMLD) and x-ray Magnetic Circular Dichroism (XMCD) magnetometry by using the beam line 4.0.2 at Advanced Light Source of Lawrence Berkeley National Laboratory. The sample was first cooled down under a static magnetic field (4000 Oe) along [-112] direction from room temperature (RT) to 78 K. To detect antiferromagnetic (AFM) spin order in the CoO layer, a single AFM domain is required. Thus we applied a static magnetic field (400 Oe) along [-112] direction to drive YIG into a single domain state, so that single AFM domain was formed in the CoO layer due to the YIG/CoO interfacial coupling. x-ray absorption spectra (XAS) were recorded at CoO L3 edge with the normal incident geometry (x-ray beam along [111] direction) and two different x-ray linear polarizations (E//[-112] and E//[110]). A tiny XMLD effect was observed at multiple peaks ($h\upsilon_2$=778.2 eV and $h\upsilon_3$=778.7 eV) of the CoO L3 edge, where XAS intensities at $h\upsilon_2$=778.2 eV and $h\upsilon_3$=778.7 eV (I_2 and I_3) deviate oppositely when turning the x-ray linear polarization from y[-112] direction ($\phi = 0°$) to [110] direction ($\phi = 90°$) (Fig. 1**b**). To study AFM spin order in the CoO layer quantitatively, we define R_{L3} as the ratio of XAS intensity at $h\upsilon_2$=778.2 eV and $h\upsilon_3$=778.7 eV ($R_{L3} = I_2/I_3$).

R_{L3} was recorded with different x-ray linear polarizations varying from $\phi = 0°$ to $90°$. As shown in Fig. 1**c**, ϕ dependence of R_{L3} is well fitted by $R_{L3} = A\cos^2\phi + B$ and the magnitude of the XMLD effect (ΔR_{L3}) can be extracted by this fitting. There are two major contributions to XMLD in single-crystalline CoO, AFM spin order and a crystal field (magnetostriction effect). In order to pick up the contribution from AFM spin order, temperature dependence of ΔR_{L3} is measured in this sample (Fig. 1**d**). ΔR_{L3} monotonically decreases when temperature changes from 78 K to 200 K, and the change is saturated above 200 K. The temperature-independent value is the contribution from a crystal field. We have fitted the temperature-dependent ΔR_{L3} in Fig. 1**d**, using a expression for $< M^2 >$.[1,2] As a

result, Néel temperature is estimated to be $T_{\mathrm{N}} = 208 \pm 10$ K for the 6 nm-thick CoO layer in this sample.

The CoO layer was studied also by XMCD. During the measurement, a static magnetic field (500 Oe) was applied along the x-ray beam direction deviated from the sample surface by 20°. XAS of the CoO layer was taken with right-circular polarized (RCP) and left-circular polarized (LCP) x-ray lights. As shown in Fig. 2**a**, no XMCD signal appears at Co L_3 and L_2 edges. As reported by literatures,[3] Co films usually generate a XMCD signal by around 6.5%, which corresponds to the total magnetic moment of 1.6 μ_{B} per Co atom. Thus uncompensated Co spins in the CoO layer is estimated to be less than 0.005 μ_{B} per a Co atom, by which the CoO layer is confirmed to be antiferromagnetic; uncompensated Co spins are negligiblely small.

Supplementary Note 2: temperature dependence of ISHE signals excluded the direct coupling at the YIG/AFM interface

Temperature dependent of the exchange coupling between the magnetic $Y_3Fe_5O_{12}$ and an antiferromagnet can be a possible reason for the peak feature of our spin pumping signal. To exclude this direct coupling, a 5 nm-thick Cu layer was inserted between the magnetic $Y_3Fe_5O_{12}$ and CoO (or NiO) layer. In the Fig. 3, we show the temperature dependence of V_{ISHE} for the $Y_3Fe_5O_{12}$/Cu/CoO/Pt tetralayer film (**a**) and the $Y_3Fe_5O_{12}$/Cu/NiO/Pt tetralayer film (**b**). In the both samples, similar peak feature can be observed, which suggests that the direct exchange coupling between $Y_3Fe_5O_{12}$ and an antiferromagnet is not necessary for the V_{ISHE} enhancement around Néel temperatures. We also noticed that the peak positions and peak shapes of those tetralayer films are slightly different from trilayer films, which are because of different crystal quality of the CoO (or NiO) on a $Y_3Fe_5O_{12}$ layer

and a Cu layer.

Supplementary Note 3: microwave frequency dependence of ISHE signals for various systems

In a $Y_3Fe_5O_{12}$ (3 μm)/NiO (1.5 nm)/Pt (10 nm) trilayer film, where an antiferromagnetic NiO layer is inserted between the $Y_3Fe_5O_{12}$ and the Pt layers, microwave-frequency dependence of V_{ISHE} was found to exhibit a similar behavior to that in the $Y_3Fe_5O_{12}$/CoO/Pt trilayer film (Fig. 4**a**). The strongest frequency (f) dependence of V_{ISHE}/P_{ab} appears at T = 280 K, at which V_{ISHE} also gets maximized. The similar f dependence observed in the two antiferromagnetic systems implies that it is a universal feature of spin pumping across an antiferromagnet.

In contrast, microwave-frequency dependence of V_{ISHE} for a $Y_3Fe_5O_{12}$ (3 μm)/Pt (10 nm) bilayer film, shown in Fig. 4**b**, does not exhibit strong f dependence at all the temperatures, being consistent with the conventional spin pumping theory.

Supplementary References

(1) Alders, D. et al. Temperature and thickness dependence of magnetic moments in NiO epitaxial films. Physical Review B **57**, 11623 (1998).

(2) Scholl, A., Stöhr, J., Lüning, J. & Seo, J. W. Observation of antiferromagnetic domains in epitaxial thin films. Science **287**, 1014 (2000).

(3) GALERA, R. M. et al. Magnetic and Superconducting Materials (World Scientific Publishing Company, 2000).
